# A simple decision to move in response to touch reveals basic sensory memory and mechanisms for variable response times

**DOI:** 10.1113/JP276356

**Published:** 2018-09-19

**Authors:** Stella Koutsikou, Robert Merrison‐Hort, Edgar Buhl, Andrea Ferrario, Wen‐Chang Li, Roman Borisyuk, Stephen R. Soffe, Alan Roberts

**Affiliations:** ^1^ School of Biological Sciences University of Bristol 24 Tyndall Avenue Bristol BS8 1TQ UK; ^2^ Medway School of Pharmacy University of Kent Anson Building, Central Avenue Chatham Maritime ME4 4 TB UK; ^3^ School of Computing Electronics and Mathematics University of Plymouth Drake Circus Plymouth PL4 8AA UK; ^4^ School of Psychology and Neuroscience University of St Andrews 9 South Street St Andrews Fife KY16 9JP UK

**Keywords:** Decision‐making, Xenopus laevis, Reticulospinal neurons, Locomotion, Somatosensory

## Abstract

**Key points:**

- Short‐term working memory and decision‐making are usually studied in the cerebral cortex; in many models of simple decision making, sensory signals build slowly and noisily to threshold to initiate a motor response after long, variable delays.
- When touched, hatchling frog tadpoles decide whether to swim; we define the long and variable delays to swimming and use whole‐cell recordings to uncover the neurons and processes responsible.
- Firing in sensory and sensory pathway neurons is short latency, and too brief and invariant to explain these delays, while recordings from hindbrain reticulospinal neurons controlling swimming reveal a prolonged and variable build‐up of synaptic excitation which can reach firing threshold and initiate swimming.
- We propose this excitation provides a sensory memory of the stimulus and may be generated by small reverberatory hindbrain networks.
- Our results uncover fundamental network mechanisms that allow animals to remember brief sensory stimuli and delay simple motor decisions.

**Abstract:**

Many motor responses to sensory input, like locomotion or eye movements, are much slower than reflexes. Can simpler animals provide fundamental answers about the cellular mechanisms for motor decisions? Can we observe the ‘accumulation’ of excitation to threshold proposed to underlie decision making elsewhere? We explore how somatosensory touch stimulation leads to the decision to swim in hatchling *Xenopus* tadpoles. Delays measured to swimming in behaving and immobilised tadpoles are long and variable. Activity in their extensively studied sensory and sensory pathway neurons is too short‐lived to explain these response delays. Instead, whole‐cell recordings from the hindbrain reticulospinal neurons that drive swimming show that these receive prolonged, variable synaptic excitation lasting for nearly a second following a brief stimulus. They fire and initiate swimming when this excitation reaches threshold. Analysis of the summation of excitation requires us to propose extended firing in currently undefined presynaptic hindbrain neurons. Simple models show that a small excitatory recurrent‐network inserted in the sensory pathway can mimic this process. We suggest that such a network may generate slow, variable summation of excitation to threshold. This excitation provides a simple memory of the sensory stimulus. It allows temporal and spatial integration of sensory inputs and explains the long, variable delays to swimming. The process resembles the ‘accumulation’ of excitation proposed for cortical circuits in mammals. We conclude that fundamental elements of sensory memory and decision making are present in the brainstem at a surprisingly early stage in development.

## Key points

- Short‐term working memory and decision‐making are usually studied in the cerebral cortex; in many models of simple decision making, sensory signals build slowly and noisily to threshold to initiate a motor response after long, variable delays.
- When touched, hatchling frog tadpoles decide whether to swim; we define the long and variable delays to swimming and use whole‐cell recordings to uncover the neurons and processes responsible.
- Firing in sensory and sensory pathway neurons is short latency, and too brief and invariant to explain these delays, while recordings from hindbrain reticulospinal neurons controlling swimming reveal a prolonged and variable build‐up of synaptic excitation which can reach firing threshold and initiate swimming.
- We propose this excitation provides a sensory memory of the stimulus and may be generated by small reverberatory hindbrain networks.
- Our results uncover fundamental network mechanisms that allow animals to remember brief sensory stimuli and delay simple motor decisions.

## Introduction

In response to sensory stimulation, most animals, from crayfish to man, take 50–200 ms to make a coordinated motor response like swimming or moving their eyes (Yerkes, 1904; Mcgill & Gibbon, 1965; Fuchs, 1967; Reichert & Wine, 1983; Domenici & Batty, 1994). Current hypotheses for the decision making processes in higher brain regions of humans and other mammals are based on correlations of neuron firing in the motor cortex (Hanes & Schall, 1996; Schall, 2003; Smith & Ratcliff, 2004). They propose a summation of noisy excitation towards a threshold as sensory information is accumulated or ‘integrated’ (Carpenter & Williams, 1995; Gold & Shadlen, 2007; Brody & Hanks, 2016; Noorani & Carpenter, 2016). To understand the origin of such delays and their variability, the step‐by‐step neuronal pathway from stimulus to response needs to be traced. What is the activity in the sensory pathways? Where are brief sensory stimuli held and remembered? Can we find the accumulator neurons where excitation summates to threshold? Primate cortical circuits are very complex (Hanes & Schall, 1996; Glimcher, 2003; Smith & Ratcliff, 2004), so simpler systems are needed to make analysis of the cellular mechanisms for the decision‐making process possible. All animals make very simple, basic decisions about whether and how to respond to external stimuli (Kristan, 2008). In young frog tadpoles, we already have some evidence suggesting that accumulation of excitation in reticulospinal neurons precedes initiation of swimming in response to touch, and that latencies can be long (Buhl *et al*. 2015). We explore the details of this process and the mechanisms responsible for long and variable delays in the decision to swim.

Hatchling *Xenopus* tadpoles can start to swim in response to skin touch stimuli. Paired whole‐cell recordings have been used to obtain remarkably detailed information on the identity, properties and connections of many of the neurons in the pathways initiating and controlling swimming (Roberts *et al*. 2010; Li, 2011). These include the primary touch sensitive sensory neurons and the sensory pathway neurons projecting to the brain. Critically, we have characterised the reticulospinal hindbrain ‘descending interneurons’ (hdINs), which drive spinal neurons during swimming and are probable homologues of brainstem neurons in zebrafish larvae, necessary for swimming rhythm generation (Kimura *et al*. 2013; Ljunggren *et al*. 2014). Following a skin stimulus, whole‐cell recordings show that excitation builds up in hdINs until firing threshold is reached and swimming starts (Li *et al*. 2004, 2009; Buhl *et al*. 2015). We have concluded that the hdINs are where the decision to swim is made. The pathways from head skin touch to excitation of hdINs on the same side of the body, via a special group of trigeminal interneurons, are defined (Buhl *et al*. 2012). However, it remains unclear how a brief sensory stimulus can lead to the longer delays before hdINs fire and swimming starts to stimulation on the opposite side of the body.

Our aim here is to understand how variable delays to the start of swimming can result from a transient skin sensory stimulus. We simplify the problem by only considering the case when swimming starts on the opposite side to a skin stimulus and do not examine how the tadpole normally ‘decides’ which side should start first. Focusing on brief current pulse stimuli to the trunk skin, we first define response times by measuring delays to the first bend of swimming, and the first ventral root burst in immobilised animals, and show that they are long and variable. Whole‐cell recordings then show that firing in the sensory and sensory pathway neurons is too brief to explain longer delays. We therefore analyse a variable pattern of summating excitatory postsynaptic potentials (EPSPs) in reticulospinal hdINs when the trunk skin on the opposite side is stimulated. These EPSPs can depolarise hdINs for nearly 1 s and, if large enough, can reach the hdIN firing threshold and lead to firing and the start of swimming. To generate this persistent pattern of excitation in hdINs, the brief sensory stimulus must be remembered by other neurons presynaptic to the hdINs whose firing extends the sensory signal. By building models of neuron networks that excite hdINs we investigate one possibility and show that inserting a small recurrent excitatory network in the brainstem sensory pathways can extend firing, produce noisy summating excitation of hdINs and generate long and variable delays in their firing. We conclude that long‐duration excitation of hdINs, presumably produced by extended firing of brainstem neurons, provides a basic sensory memory of the brief stimulus (Wang *et al*. 2013). The hdINs in the hindbrain which drive swimming are the location where excitation is slowly accumulated to firing threshold, and where a simple decision to swim is made. The elements of a basic working memory process and a decision mechanism based on an accumulation of excitation to threshold (Carpenter & Williams, 1995; Gold & Shadlen, 2007; Brody & Hanks, 2016; Noorani & Carpenter, 2016) are therefore present at a surprisingly early stage in brain development in a basal vertebrate.

## Methods

### Ethical approval

Procedures for obtaining developmental stage 37/38 *Xenopus laevis* tadpoles (Nieuwkoop & Faber, 1956) complied with UK Home Office regulations. All experimental procedures on stage 37/38 tadpoles are unregulated but were approved after local ethical committee review. We confirm that our work complies with the ethical principles under which *The Journal of Physiology* operates. Experiments were performed at 18–22°C.

### Behaviour

High speed movies of tadpole responses in tap water were taken at 300 frames/s using a Casio EX‐F1 camera. The tadpole was supported dorsal side up by two pins on either side of its neck region. The pins were inserted at an angle into the Sylgard base of a Petri dish so they formed a ‘V’. The dish was illuminated by diffuse LED light from below. A 5 ms pulse powered an LED in the video frame to show the time of a current pulse stimulus (5 ms; 9 V) delivered via a bipolar electrode (insulated except at the tip and ∼0.1 mm diameter) held by hand in contact with the tadpole's trunk skin near the level of the anus. The stimulus voltage and duration was adjusted to give swimming responses in ∼50% of trials. In videos, response times were calculated as the number of frames between the LED flash and the first flexion of the body times the frame interval (3.33 ms at 300 frames/s). Response times were also measured using a photo‐transducer, where a bright point source LED was positioned 2 cm above the tadpole so its enlarged shadow fell on an optical gradient placed above a photovoltaic cell. The photocell output changed as the shadow of the tadpole moved to the left or right along the optical gradient. Output was displayed, and the delay time to the first flexion measured, using a PC oscilloscope (Picoscope; Pico Technology, St Neots, UK).

### Electrophysiology

Motor nerve recordings of fictive swimming, alone or in combination with whole‐cell recordings in bridge mode, were performed in immobilised *Xenopus laevis* stage 37/38 tadpoles using methodology described extensively elsewhere (Li *et al*. 2001, 2010; Lambert *et al*. 2004; Buhl *et al*. 2015). The criteria for identification of hdINs were: a less negative resting potential (−50.9 ± 4.9 mV), fires a single, broad spike (∼2 ms at 0 mV) to depolarising current, fires one spike reliably on each swimming cycle and has a ventral soma with descending axon (Li *et al*. 2007b).

### Modelling methods

To investigate the ability of a small population of recurrently connected neurons to generate variable delays in response to brief input we built a computational model of 30 ‘hindbrain extension’ neurons (hexNs). Each hexN consisted of two electrically connected compartments, one representing the combined dendrites and soma and the other representing the axon. The equations governing the dynamics of these compartments were based on the Hodgkin‐Huxley equations, but with the membrane properties of an unspecialised, generic tadpole neuron (spinal motoneuron; Sautois *et al*. 2007). The parameters for the dendrite/soma and axonal compartments were identical, except that the maximum conductance values of all active channels (Na, K_fast_, K_slow_) were increased by a factor of five in the axonal compartment. The total capacitance of each compartment was 5 pF, and the inter‐compartment conductance was 10 nS. We used a two‐compartment model because random networks of single compartment neurons with motoneuron properties were not able to produce persistent rhythmic firing when coupled by glutamatergic synapses with NMDA receptors. During strong excitatory synaptic input, the neurons in such networks became very depolarised and stopped firing because of depolarisation block. A more realistic model incorporating a second compartment representing the axon did not have this problem; when the soma/dendrite compartment was depolarised by excitatory synaptic input the axonal compartment could continue to spike repetitively.

The 30 neurons in the hexN population were connected together by excitatory glutamatergic synapses. The number of other hexNs that a given hexN received synaptic inputs from was chosen by sampling (and rounding down) a normally distributed random variable with μ, σ = 3.0. Each set of presynaptic neurons was chosen randomly, with no self‐connections or multiple connections between the same pair.

Synapses were implemented using the same equations and parameters as previously (Roberts *et al*. 2014). Specifically, when the membrane potential of the presynaptic neuron's axonal compartment crossed 0 mV, AMPA and NMDA receptors in the postsynaptic neuron's dendrite/soma compartment were activated. The maximum conductances of AMPAR and NMDAR channels at hexN–hexN synapses were 1.5 and 1.8 nS, respectively.

We hypothesise that following skin stimulation the population of hexNs receives transient excitatory input from sensory pathway dorsolateral commissural neurons (DLCs). The model included a population of 30 sensory pathway DLC input neurons, each of which spiked once at the beginning of a simulation. The nearly synchronous DLC spike times were generated by repeatedly sampling from a normal distribution with a mean of 5 ms and standard deviation of 2 ms, until values greater than 5 ms were obtained. This gave a pattern of firing similar to that of tadpole DLC neurons (Fig. 2; Li *et al*. 2003). After preliminary trials the DLC input neurons were connected to the hexNs randomly, with a probability of 0.4. These connections were chosen randomly once and then kept the same across all simulations of one network. Input neuron to hexN synapses activated a mixture of AMPA and NMDA receptors, with maximum conductances of 1.1 and 1.4 nS, respectively.

To introduce trial‐to‐trial variability, the strength of DLC–hexN and hexN–hexN synapses varied across simulations. Specifically, the maximum conductance of each synapse was scaled down by a randomly chosen value (0.8, 0.6, 0.4, 0.2 or 0) at the start of each simulation, with the scaling factor for each synapse chosen independently. In later trials, we also made synaptic strength vary randomly with each occurrence of a presynaptic spike. After any spike event, the synaptic strength of connected neurons was scaled down by a randomly chosen value (0.8, 0.6, 0.4, 0.2 or 0). This variability mimicked the unreliable nature of individual synapses observed in the *Xenopus* spinal cord (Li *et al*. 2002, 2003, 2006, 2007a; Buhl *et al*. 2012). In some experiments (including those shown in Fig. 5) we also varied DLC to hexN synapse strengths from trial to trial using the same approach. However, we found that this had little effect on the variability of the network's output; similar results could be achieved by uniformly scaling down DLC to hexN connection probability and synapse conductances by a fixed amount.

We used two methods to study how the output of the hexN network could affect downstream reticulospinal hdINs. Initially, we connected a random subset of hexNs (with a connection probability of 0.2) to five model hdINs (with no connections between them). The model used for these hdINs was described previously (Roberts *et al*. 2014). The hexN spikes activated AMPA and NMDA receptor channels with maximum conductances of 0.25 and 0.1 nS, respectively.

To study in more detail how the activity in the hexNs can produce excitation which accumulates and leads to firing in a population of reticulospinal hdINs, we used another, more biologically realistic, model of 30 multi‐compartmental hdINs that were connected to each other via glutamatergic synapses and gap junctions and described in Hull *et al*. (2016). Each hdIN in this model population received synaptic input at the firing times of three randomly selected hexNs from the hexN network model. These inputs activated AMPA and NMDA receptors in the hdIN's soma/dendrite compartment with maximum conductances of 0.125 nS for AMPA and 0.15 nS for NMDA.

All simulations were performed using NEURON 7.3 (Carnevale & Hines, 2006) with Python 2.7. The simulations of the hexN network were built using the PyNN library (Davison *et al*. 2008) and the spike times of these hexNs were then passed into the hdIN network simulation, which was built using the Morphforge library (Hull & Willshaw, 2014) using NEURON as the simulator. The simulation time step was 0.025 ms for the hexN network simulations and 0.1 ms for the multi‐compartmental hdIN network simulations. Model source code is available from ModelDB (http://modeldb.yale.edu/).

### Experimental design and statistical analysis

Experimental data were analysed using MS Excel and Minitab (version 13; Minitab Inc.), including use of some purpose written routines. Most experimental values were non‐normal and so, unless stated otherwise, are expressed as median with interquartile range (IQR). To compare variability between measurements of response delay for first movement (from video recordings) and spike delays (from electrical recordings of sensory, sensory pathway and excitatory reticulospinal neurons), coefficients of variability (CV = mean/standard deviation) were calculated for each animal/neuron. CV data were normalised by log transformation and a generalised linear model was then used with Tukey's pairwise comparisons to test for differences between datasets.

## Results

### Response times for tadpole swimming

**Figure 1:**
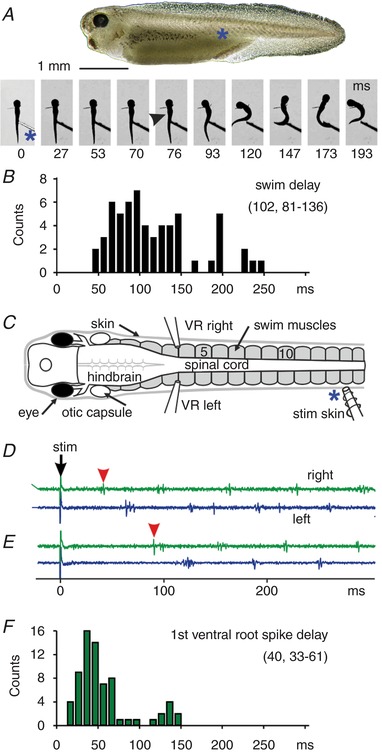
Response times to the first flexion and ventral root burst of swimming to current pulse trunk skin stimulation *A, Xenopus* tadpole with stimulus site marked (^*^) and frames from a video (stimulus at *t* = 0 ms). The tadpole (supported by pins in the neck region) flexes to unstimulated left side starting at 76 ms (arrowhead) and swims off. *B*, distribution of delays to the start of the 1st flexion of swimming. *C*, diagram of immobilised tadpole with stimulating and ventral root (VR) recording electrodes. *D* and *E*, motor nerve responses to 0.1 ms pulse to trunk skin (↓) to show when swimming started on the right, unstimulated side (red arrowheads). *F*, distribution of delays to the first ventral root spikes when swimming started on the unstimulated side. Numbers in brackets on graphs are median and interquartile range (IQR).

Touch to the trunk skin with a fine hair usually leads to tadpole swimming (Boothby & Roberts, 1995). To measure response times to the first flexion of swimming, a current pulse stimulus was given to ventral trunk skin to evoke a single spike in sensory Rohon‐Beard neurons (Clarke *et al*. 1984). Videos at 300 frames/s showed that response times were long and ranged from 63 to 160 (median 97 ms, IQR 80–113 ms; 15 trials in 3 tadpoles; Fig. 1 *A*). More extensive latency measures made using a photo‐transducer confirmed that response times were long and variable (median 102, IQR 81–136 ms; >5 trials in each of 15 tadpoles; Fig. 1 *B*). These measures suggest that delays to the start of tadpole swimming are longer and more variable than simple reflexes or the ballistic escape responses seen in fish (Korn & Faber, 2005). Variability in the location and closeness of the stimulating electrode to the skin could make some contribution to variability in response delays.

### Response times for swimming in immobilised tadpoles

Motor nerve recordings from immobilised tadpoles were used to define delays to the start of fictive swimming following a threshold (0.1 ms current pulse) stimulus to the left trunk skin (Fig. 1 *C*–*F*). Swimming, with characteristic left–right alternation and head to tail progression, started on the unstimulated (right) side in 68.4% of 114 trials (19 tadpoles). The median delay to the first motor burst on the unstimulated side was 40 ms (IQR 33–61; Fig. 1 *F*). These delays to the start of fictive swimming, when the CNS is exposed to physiological saline, were shorter than delays measured behaviourally (Fig. 1 *B*). However, both were long and variable compared to *Xenopus* motoneuron firing delays in a simple flexion reflex to a trunk skin stimulus (7.0–13.8 ms; Li *et al*. 2003).

### Can firing in the sensory pathway explain response delays and their variability?

The tadpole's skin is innervated by touch‐sensitive trigeminal sensory neurons in the head, and spinal Rohon‐Beard (RB) neurons in the trunk (Roberts *et al*. 2010). Both types of sensory neuron fire a single action potential in response to a <1 ms current pulse in their receptive field (Clarke & Roberts, 1984; Buhl *et al*. 2012). Head skin sensory neurons excite trigeminal sensory pathway neurons in the hindbrain and also the dorsolateral commissural sensory pathway neurons (DLCs) in the rostral spinal cord which have commissural projections (Buhl *et al*. 2012, 2015). Trunk skin sensory RB neurons also directly excite these DLC neurons which project axons into the contralateral hindbrain (Li *et al*. 2003). However, paired recording failed to find any evidence for direct synaptic excitation of reticulospinal hdIN neurons by DLC neurons (Buhl *et al*. 2015). We have focused exclusively on this commissural pathway because we are confident that DLC neurons provide the only skin sensory pathway activating swimming on the unstimulated side of the body. We have not investigated the ipsilateral pathway from the skin to the hindbrain, which is formed by the ascending axons of both sensory RB neurons and the dorsolateral ascending sensory pathway neurons which they excite (Li *et al*. 2004).

**Figure 2:**
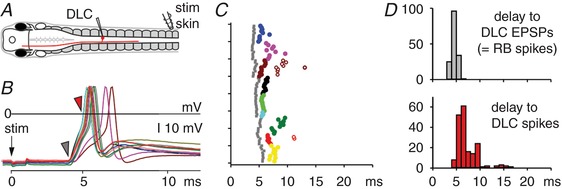
Firing times in sensory pathway DLC neurons following a trunk skin stimulus *A*, diagram to show the location of electrodes and the recorded neuron. *B*, ten superimposed responses in a DLC. Delays to the EPSP (grey arrowheads) give the sensory RB spike times and DLC spikes are clear (red arrowheads). *C*, spike time raster plots for RBs (grey squares) and DLCs (coloured circles: 1st spikes filled, 2nd/3rd spikes open; *n* = 9 DLCs). *D*, spike latency plots of RBs and DLCs.

To investigate the timing of sensory firing, whole‐cell recordings from sensory pathway DLC neurons were used to measure responses to a current pulse to the trunk skin (Fig. 2). EPSP onset latencies in DLC neurons indicated that the firing delays of the single spikes in sensory RB neurons were short and consistent (mean 4.7 ± 0.6 ms; range 3.6–6.3 ms; 156 trials in 9 DLCs). The DLC spike latencies were a little longer and more variable (mean 6.5 ± 1.1 ms, range 4.8–10.8 ms for 1st spike; overall range 4.8–16.4 ms). The latency and variability of firing times in these sensory and sensory pathway neurons cannot explain the length and variability of response times to the start of swimming (Fig. 1 *F*). We therefore recorded from the hindbrain neurons that drive swimming to examine the pattern of synaptic input that leads to their firing and the start of swimming following a sensory stimulus to the skin.

### The role of reticulospinal hdIN neuron firing in swimming

**Figure 3:**
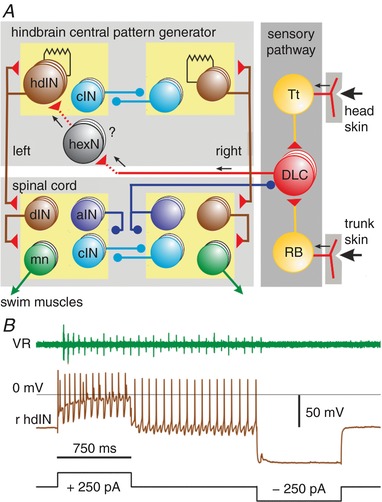
The tadpole swimming network and role of reticulospinal hdIN neurons *A*, diagram of the touch sensory pathways to the opposite side from the head and trunk skin, and the hindbrain and spinal neurons controlling swimming. Tt, trigeminal touch sensory; RB, Rohon‐Beard touch sensory; DLC, dorsolateral commissural sensory pathway; hexN, hindbrain extension neurons; hdIN, hindbrain descending interneurons; cIN, reciprocal inhibitory commissural interneurons; aIN, recurrent inhibitory ascending interneurons; mn, motoneurons. Red triangles are glutamatergic excitatory synapses. Blue circles are glycinergic inhibitory synapses. Synapses onto a box connect to all neurons in the box. *B*, recording from a right hdIN where activity in the whole swimming network (seen in left ventral root (VR): green trace) could be initiated by positive current and terminated by negative current into this single neuron. The right hindbrain was transected just caudal to the otic capsules (see Fig. 1 *C*).

Whole‐cell recordings from hindbrain descending interneurons (hdINs) have been used in our detailed studies of their properties and key role in the swimming network (Fig. 3 *A*; Li *et al*. 2006, 2010; Soffe *et al*. 2009). We have concluded that the hindbrain populations of ∼30 hdINs on each side are equivalent to reticulospinal neurons and that they are critical for the initiation and generation of swimming. We need to summarise the existing evidence on these hdINs before considering the form and significance of their responses to skin stimulation (see Roberts *et al*. 2010; Li, 2011). Overall, hdINs form the hindbrain part of a longitudinal column of relatively ventral cells extending through hindbrain and into spinal cord, with dendrites in the marginal zone and an ipsilateral descending axon. Many of those in the hindbrain (hdINs) also have an ascending axon. They have diagnostic physiological features: a long duration action potential and only single spike firing to depolarising current; rebound single‐spike firing following hyperpolarisation, but only when depolarised; and electrical coupling to other hdINs. They co‐release glutamate and ACh to excite each other (via AMPARs, NMDARs and nAChRs), spinal motor neurons, and reciprocal and recurrent inhibitory neurons (mainly via AMPARs). Perfusion of NMDA gates in hdIN pacemaker firing in the normal swimming frequency range. When swimming is initiated the hdINs on one side fire a single spike (synchronised by their gap junction coupling) immediately before the start of ventral root activity. They then fire reliably once on each swim cycle immediately before all other swim neurons (Soffe *et al*. 2009). When hdINs fire, their mutual glutamate synaptic excitation summates to sustain swimming. Firing within the hdIN population is synchronised by their electrical coupling (Li *et al*. 2009; Hull *et al*. 2016). Current injection into a single hdIN can speed up, slow down or stop swimming (Li & Moult, 2012; Moult *et al*. 2013), and in some cases can be sufficient to start swimming (Fig. 3 *B*; *n* = 12). Together, these experiments provide evidence for the critical role played by hdINs in the initiation of swimming.

The hdINs therefore initiate swimming following sensory stimulation (Buhl *et al*. 2012, 2015) but also sustain it through their mutual electrical and chemical excitation (Hull *et al*. 2016), cellular and pacemaker properties, and rebound from reciprocal inhibition between the two sides. These critical roles made the reticulospinal hdINs the key neurons for us to examine to try to account for the long and variable delays to the start of swimming.

### Variability in reticulospinal hdIN neuron firing delays in response to skin stimulation

**Figure 4:**
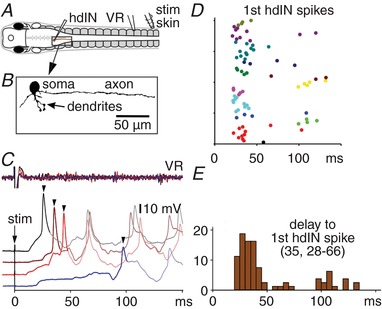
Responses of reticulospinal hdIN neurons to contralateral trunk skin stimulation *A*, diagram to show the location of electrodes and the recorded hdIN neuron. *B*, anatomy of hdIN revealed by neurobiotin filling. *C*, examples of 4 hdIN responses to trunk skin stimulus. Arrowheads mark 1st spike. *D* and *E*, hdIN 1st spike time raster plot and 1st spike delays (*n* = 80 trials in 13 hdINs).

We used whole‐cell recordings of 17 hdINs from locations close to where their firing is earliest during each cycle of swimming activity (Soffe *et al*. 2009) to define responses to a contralateral trunk skin stimulus. If this stimulus was sufficient, the hdINs were depolarised to threshold, fired, and swimming was initiated (Fig. 4). For 117 responses recorded in seven hdINs on the same side as a ventral root (VR) recording, the first dIN spike almost exclusively preceded the first VR burst on the same side, whether swimming started on that side (69/70 = 98.6% of responses) or the opposite side (48/51 responses = 94.1%). As expected from the delays to the start of swimming, delays to the first spikes in hdINs were also long and variable (median 35.4 ms, IQR 27.8–65.7; Fig. 4 *C*–*E*).

To compare relative degrees of variability in the pathway from touch to swimming, we calculated coefficients of variability (CV = SD/mean) for each neuron/animal for activity at different stages. Firing in hdINs (CV = 0.36 ± 0.29) was significantly more variable in delay than for either RBs or DLCs (CVs = 0.04 ± 0.02 and 0.07 ± 0.06, respectively; *P* < 0.001 in each case; GLM with Tukey's *post hoc* comparison on log‐transformed data). Variability in delay to the first swimming movement (CV = 0.37 ± 0.18) was also significantly greater than for RB or DLC firing (*P* < 0.001) but no different to that for hdIN firing (*P* = 0.95).

### Synaptic excitation of reticulospinal hdIN neurons following trunk skin stimulation

**Figure 5:**
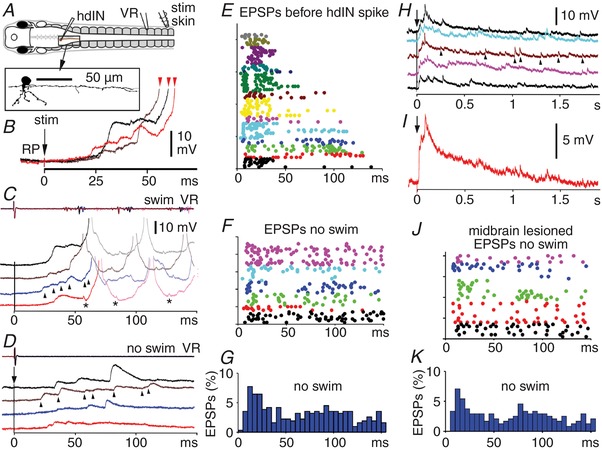
Summation of EPSPs to firing threshold in reticulospinal hdINs following a trunk skin stimulus on the opposite side *A*, neurobiotin fill of the neuron in the left caudal hindbrain recorded in *B*–*D* (box location as in Fig. 4 *A*.) *B*, three superimposed responses (from *C*) to contralateral stimuli evoking swimming show the noisy rise of excitation towards spike threshold (spike onsets marked by red arrowheads). *C* and *D*, recordings show variability in EPSP summation in responses to trunk skin stimulation just above (*C*) and below (*D*) swim threshold (see ventral root (VR)). Arrowheads mark example EPSPs and responses are offset for clarity. Asterisks mark artifacts due to spikes in another hdIN recording electrode on the other side. *E* and *F*, raster plots of EPSP latencies in response to skin stimuli at *t* = 0 where each colour is a different hdIN and each row is a different response. *E*, EPSPs up to the time of the first hdIN spike of swimming (14 hdINs). *F*, EPSPs to stimuli below swimming threshold (6 hdINs), persist for more than 150 ms after the stimulus. *G*, EPSP latency distributions for responses in *F*. *H*, slower time scale recordings show long duration of responses below swim threshold. Arrowheads mark example EPSPs. *I*, the long duration of the excitation is clear from an average of the 5 responses in *H*. *J* and *K*, raster plots and distributions of EPSP latencies, as in *F* and *G*, in animals with the midbrain removed.

When the trunk skin is stimulated, hdINs receive excitation in the form of a very variable series of summating EPSPs which can depolarise the hdINs to firing threshold (Fig. 5 *A*–*E*). Variability in the summation was reflected in a lack of relationship between the latency of the first EPSP of a response (median 8.7 ms, IQR 6.9–12.6) and the time of the first spike (see above; linear regression *R* ^2^ = 0.00; *n* = 83 responses in 14 hdINs; data not shown). There could also be a long delay to the first large EPSP. The summating EPSPs normally led to a final ramp rising to threshold and firing (Fig. 5 *B*). It seems unlikely that background synaptic input contributes significantly to this summation and the firing time of hdINs because, at rest, hdINs were surprisingly quiescent (hdINs do not fire at rest) and received little spontaneous synaptic input. The resting membrane potential fluctuated by only 0.44 ± 0.14 mV (mean of SDs for eighteen 1 s samples in 6 hdINs). Instead, variability of summation must result from the pattern of input following a skin sensory stimulus.

The individual EPSPs also varied in amplitude (overall range 0.1–13.5 mV; median 2.3 mV, IQR 1.2–4.1 mV, *n* = 243 EPSPs in 57 responses where hdINs fired, in 12 animals; estimated as the peak change in membrane potential between the starts of successive EPSPs). These EPSPs could each result from single presynaptic spikes and all lay within the large range of EPSP amplitudes previously recorded from tadpole neuron pairs (Li *et al*. 2003, 2006). However, some larger EPSPs could also result from near‐synchronous spikes, occurring occasionally by chance in presynaptic neurons which individually produce smaller EPSPs.

Measurements above were made of EPSP latencies in the period before hdIN firing and the start of swimming (Fig. 5 *C* and *E*). However, when hdIN depolarisation failed to reach threshold and evoke firing or swimming, there was still a variable and extended pattern of EPSPs with a peak of occurrences between 10 and 25 ms (Fig. 5 *D, F* and *G*), but persisting much longer. Following such subthreshold skin stimulation, hdINs usually remained depolarised for more than a second (well beyond the range plotted in Fig. 5 *F* and *G*) and throughout this time, distinct EPSPs were still visible (Fig. 5 *H; n* = 46 responses in 6 hdINs). Averaging of slower time scale recordings showed the broad form of the depolarisation, which reached a peak at around 50–100 ms and had a mean half‐fall time of 158 ms (SD 42, 5 measures in 5 dINs; Fig. 5 *I*).

The dispersed pattern of summating EPSPs that we have recorded in hdINs cannot be explained by the short latency, single firing patterns of the sensory and sensory pathway neurons (Fig. 2). Instead, the timing of these EPSPs provides clear evidence that there are excitatory neurons, presynaptic to the hdINs, which are excited by a brief sensory stimulus but fire later, and can fire for much longer than the sensory pathway neurons described above.

Since sensory pathway DLC axons project into the midbrain it was possible that the summating hdIN EPSPs came from midbrain neurons projecting caudally into the hindbrain. However, after transverse section between the hindbrain and midbrain, hdIN responses to trunk skin stimulation were very similar to control animals. Summating EPSPs were still present and could lead to hdIN firing. EPSP amplitudes were similar to those in intact animals (overall range 0.3–10.7 mV; median 2.1 mV, IQR 1.2–4.0 mV; *n* = 123 EPSPs in 24 responses in 5 animals). When skin stimuli were below the swim threshold, the distributions of EPSPs were also very similar (Fig. 5 *J* and *K*). This suggests that the EPSPs primarily come from hindbrain neurons.

### Synaptic excitation of reticulospinal hdIN neurons following head skin stimulation

**Figure 6:**
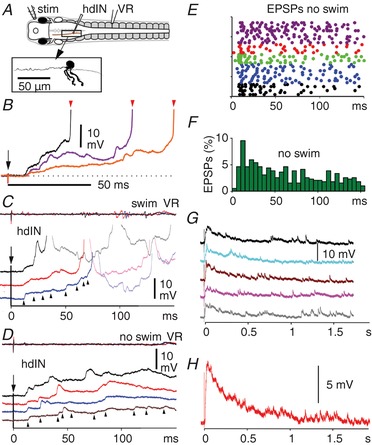
Summation of EPSPs to firing threshold in reticulospinal hdINs following a head skin stimulus on the opposite side *A*, diagram to show the location of electrodes. The inset is the hdIN neuron recorded in *C* and *D*. *B* and *C*, responses of hdINs to head skin stimulation (at arrow). *B*, three superimposed responses from different neurons leading to firing (red arrowheads). Dotted line shows resting potential. *C* and *D*, responses of hdIN in *A* to stimuli above (*C*) and below (*D*) threshold for the hdIN spike and swimming (see VR traces at top of panels) Arrowheads mark example EPSPs. *E*, raster plot of EPSP latencies from 5 hdINs (different colours) over the first 150 ms after the stimulus when swimming did not occur. *F*, EPSP latency distributions for responses in *E*. *G*, slower time scale recordings of responses below swim threshold show the prolonged responses in another hdIN. *H*, an average of responses in *G*.

Head skin stimulation can also initiate swimming starting on the unstimulated side after long, variable delays (median 35 ms; IQR 29–44) (Boothby & Roberts, 1995; Buhl *et al*. 2012, 2015). Recordings from hdINs on the unstimulated side again showed a variable pattern and distribution of summating EPSPs (5 hdINs in 4 animals; Fig. 6; see also Figs 4 and 7*G* in Buhl *et al*. 2015). The delays to the earliest EPSP were similar to those following trunk skin stimulation (median 10.2 ms, IQR 9.1–11.4; *n* = 31 responses). Stimuli subthreshold for swimming, again evoked an extended depolarisation of summating EPSPs, rising between 25 and 150 ms and varying in shape, duration and amplitude (Fig. 6 *D*–*F*). As in responses following subthreshold trunk skin stimuli, hdINs usually remained depolarised for more than a second with distinct EPSPs still visible throughout. Averages showed the depolarisations had a peak at around 50–100 ms and a mean half‐fall time of 272 ms (SD 84, 5 averages measured in 5 dINs; Fig. 6 *G* and *H*). This suggests that the same, or equivalent neurons and mechanisms are involved in extending the effect of sensory stimuli to the head and trunk skin. Since rostral DLC sensory pathway neurons are excited by both head and trunk skin stimuli (Buhl *et al*. 2015), it seems certain that they will, in turn, excite the same hindbrain neurons on the opposite side in both cases.

### Conclusion about excitation of reticulospinal hdINs

The extended pattern of summating EPSPs recorded in reticulospinal hdINs following trunk or head skin stimulation and the minimal effect of lesioning the midbrain indicates clearly the existence of further hindbrain neurons in the excitatory pathways to hdINs. As outlined above, these neurons must: fire in response to a brief skin sensory stimulus; fire later than the known sensory pathway neurons; and fire over an extended period, even in the absence of swimming; make excitatory synapses with hdINs. Firing in these neurons would act as a simple memory of the brief skin stimulus, extending the sensory signal until a decision to swim has been made, signalled by the start of firing in the hdIN population.

### A model recurrent network to extend sensory firing

**Figure 7:**
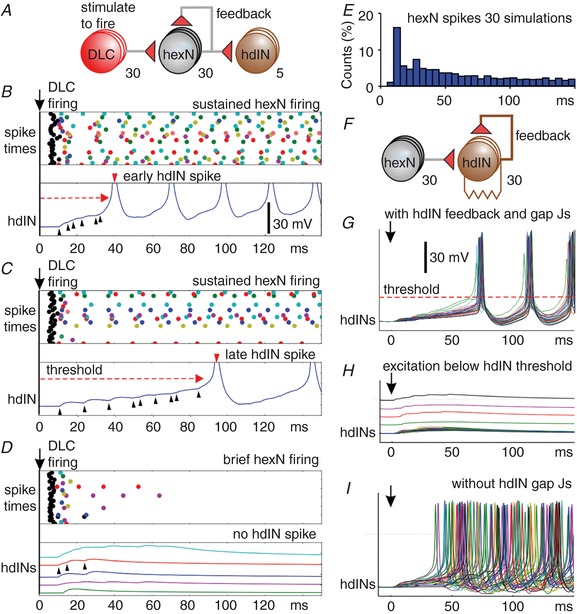
Recurrent models of reticulospinal hdIN excitation and recruitment *A*, recurrent hexN network excited by single spikes in 30 sensory pathway DLCs with 5 hdINs to monitor output. *B–D*, responses to 30 DLC spikes. Raster plots show spike times for DLCs (black) and hexNs (colours); stimulus to DLCs at arrow. Lower panels show selected hdIN voltage records. The hexNs produce variable, summating EPSPs in hdINs (black arrowheads). EPSP summation can reach threshold (dashed red line) and lead to hdIN firing (red arrowhead) after variable delays. *D*, when hexN firing is brief, EPSPs sum but do not reach hdIN firing threshold (all five traces separated for clarity). *E*, histogram of all hexN firing times in 30 trials of a single network. *F*, model of a population of 30 hdINs with electrical coupling and feedback glutamate excitation (Hull *et al*. 2015) excited by hexN spikes at times determined by the hexN recurrent network model. *G–I*, overlapped voltage records of all 30 hdINs in response to hexN excitation in one trial. *G*, excitation can sum to threshold so hdINs are recruited to spike rhythmically and almost synchronously. *H*, in another trial the EPSPs sum but do not reach hdIN firing threshold (some traces separated for clarity). *I*, without electrical coupling, hdINs fire earlier and then asynchronously. All voltage scales as in *B* and *G*.

A simple and commonly proposed mechanism to explain extended firing is that some neurons form small recurrent networks on each side of the hindbrain, lying between the sensory pathway and the hdINs (Durstewitz *et al*. 2000). To explore the plausibility of this proposal, we built a simple model network (Fig. 7 *A*) using neuron and synapse specifications from previous tadpole models (Sautois *et al*. 2007; Hull *et al*. 2016). In this network, 30 sensory pathway DLCs, with nearly synchronous spike times, produced glutamatergic excitation in a group of 30 hypothetical ‘hindbrain extension neurons’ (hexNs) with a contact probability of 0.4. To form a recurrent network, the hexNs made mutual glutamatergic synapses, activating AMPA and NMDA receptors; each hexN received input on average from three other, randomly chosen, hexNs. The synaptic strength was varied randomly between trials (scaled by 0.8, 0.6, 0.4, 0.2 or 0). Such variability has been observed in many synapses in tadpoles (Li *et al*. 2002, 2003, 2006, 2007b; Buhl *et al*. 2012). This synaptic noise was the only trial‐to‐trial variability.

In preliminary tests, we found that brief sensory DLC firing produced continuous, self‐sustained firing of all hexNs. This showed the effectiveness of recurrent excitation but did not provide significant variability. Synaptic strengths and connection probabilities of DLC to hexNs were therefore reduced until only some hexNs fired as a direct result of DLC excitation. Sustained firing was then variable across the hexN population, and on some trials hexN firing was transient (Fig. 7 *B*–*D*). These patterns of hexN firing led to slow and variable summation of EPSPs in five hdINs, connected to the hexNs to monitor their output (Fig. 7 *A*). The summating EPSPs could fail to reach hdIN firing threshold or lead to firing with variable delays (34–190 ms; *n* = 30 trials). Single hexN spikes led to small hdIN EPSPs (0.6–2.3 mV) but larger hdIN EPSPs occurred when two or more hexN spikes were nearly synchronous. Repeated trials gave data on hexN spike time distributions (Fig. 7 *E*) which, with the parameters values chosen, were broadly similar to those of EPSP times recorded physiologically in hdINs (Figs 5 *F* and 6 *F*). Further tests showed that similar hexN firing time distributions could also be produced after the random pattern of hexN‐to‐hexN synaptic contacts in the network was changed and when spike‐to‐spike variability in synaptic strength was added.

A recurrent network can therefore generate extended and variable hexN firing following transient input from the sensory pathway and produce a pattern of summating EPSPs in hdINs with which they synapsed. However, the recruitment of reticulospinal hdINs on one side of the body by hexN excitation might also be influenced by their mutual electrical coupling and excitatory synaptic connections (Li *et al*. 2009, 2010). Previous modelling showed that these connections contribute to the synchronous recruitment of rhythmic firing in the whole population (Hull *et al*. 2015). Using this previous hdIN population model with mutual electrical coupling and excitatory synaptic connections (Fig. 7 *F*; Hull *et al*. 2016), we found that the hexN firing patterns generated by our recurrent hexN network led to summation of EPSPs in a slightly different pattern in each hdIN (Fig. 7 *G*). But, if some hdINs fired, then the whole hdIN population was recruited after delays from 40 to 75 ms (10 trials). This led to a synchronised pattern of rhythmic hdIN firing like that seen during swimming. Firing was sustained by recurrent glutamate excitation within the hdIN population. This synchronised recruitment and firing depended on the electrical coupling of the hdINs because, when the hdIN electrical connections were removed, hexN input led to unsynchronised recruitment and continuous firing of the hdINs (Fig. 7 *I*).

These modelling results illustrate the plausibility of the proposal that activity in a group of neurons forming a simple recurrent network could act as a short sensory memory, extending the response to a brief sensory stimulus and providing summating excitation to produce a long and variable delay in the initiation of hdIN firing and a motor response.

## Discussion

We have shown that swimming responses to touch stimuli in young *Xenopus* tadpoles have long and variable reaction times. Recordings from hindbrain reticulospinal hdIN neurons, which play a central role in driving swimming, show a long‐lasting pattern of slowly and ‘noisily’ summating excitation following stimulation that precedes hdIN firing and the start of swimming. This pattern of excitation cannot be explained by the brief firing in sensory and sensory pathway neurons. In our data, there was ∼5 ms delay between the latest DLC spike and the earliest dIN spike recorded. This delay could certainly be explained by a direct connection, but 50% of dIN spikes were more than 20 ms after the latest DLC spike and could be up to 200 ms later. Furthermore, paired recordings from sensory pathway DLC neurons and hdINs have failed to find evidence for direct, monosynaptic synaptic excitation (Buhl *et al*. 2015). The timing of the component EPSPs of the summating excitation that leads to a hdIN spike points instead to extended firing in undefined neurons presynaptic to the hdINs. Lesions suggest that many of these neurons lie in the hindbrain. We propose that these neurons are excited by sensory pathway neurons and can extend firing for over a second. This firing, and the consequent prolonged excitation of hdINs, acts as a sensory memory of the brief stimulus which would allow temporal and spatial summation of responses to stimuli anywhere on the body surface.

The slow summation of excitation in hdINs delays their firing and the onset of swimming. Modelling shows that a population of generic neurons, with recurrent excitation and variable synaptic strengths, inserted into the sensory pathway to the hdINs, can produce such a noisy, summating pattern of excitation to dINs and lead to variable delays in their firing. The process that we have described underlying a coordinated response in a young vertebrate, just 24 h after the closure of the neural tube, bears remarkable similarities to mechanisms proposed to underlie simple decision making in the adult cortex (Wang, 2002; Gold & Shadlen, 2007). This suggests that what we have described is a fundamental feature of animal decisions to make a coordinated movement.

### How do our findings in the tadpole relate to models of decision making?

Similar neuronal events have been proposed in some of the theoretical models of the decision process leading to simple coordinated movements (Carpenter & Williams, 1995; Wang, 2002; Gold & Shadlen, 2007; Bogacz *et al*. 2010; Smith *et al*. 2011; Noorani & Carpenter, 2016). However, in many cases, the decisions involve a choice between different actions and the models involve inhibition. In the present study, we have restricted ourselves to the simplest case: the decision on whether or not to respond. In this case many models suggest that response time is determined by the noisy rise of a cumulative process, building to reach a defined voltage threshold. This is exactly what we have recorded in tadpole reticulospinal hdINs: a noisy summation of variable EPSPs builds up to spike firing threshold and leads to long and variable delays in hdIN recruitment and the start of swimming. This variability cannot be explained by the sensory input signal because we present direct evidence for a lack of suitable noise in the firing times of sensory and sensory pathway neurons. The reticulospinal hdIN populations on each side of the body therefore act as decision makers. When excitation reaches the hdIN firing threshold their whole population is recruited because they are electrically coupled (Li *et al*. 2009; Hull *et al*. 2016). This results in the excitation of motoneurons and signals the decision to swim (Buhl *et al*. 2015).

Reticulospinal systems control adult body and eye movements (Sparks, 2002; Dubuc *et al*. 2008; Jordan *et al*. 2008; Arber, 2012; Ruder *et al*. 2016) but it is the mammal cortical decision circuits, which act as higher level control systems, that have been studied in such detail (Hanes & Schall, 1996; Schall, 2003; Smith & Ratcliff, 2004). Our findings suggest that at early stages of development in tadpoles and fish (Kimura *et al*. 2013) decisions are made in reticulospinal neurons in the brainstem.

Many models of decision networks include reciprocal inhibition as there is nearly always a choice between alternatives (Bogacz *et al*. 2010; Smith *et al*. 2011; Marshall *et al*. 2012). This is also the case in the tadpole, where there is powerful reciprocal inhibition between the antagonistic motor systems on the two sides of the body (Dale *et al*. 1986). The tadpole has to decide which side to flex first (Buhl *et al*. 2015). Inhibition may well be involved in this aspect of the full network and its role in the whole decision process remains to be addressed.

### Can we explain the slow, noisy build‐up of excitation in reticulospinal neurons?

Following a brief stimulus to the skin, excitation of tadpole reticulospinal hdIN neurons, whose firing initiates swimming, far outlasts the single spikes in the sensory pathway (Roberts & Sillar, 1990; Li *et al*. 2003, 2004; Buhl *et al*. 2015). The patterns of EPSPs recorded in hdINs could be mimicked in model networks by inserting a small excitatory recurrent network, with inherent variation, into the sensory pathway to extend firing. Previous studies have proposed roles for recurrent networks in cortical working memory (Goldman‐Rakic, 1995; Durstewitz *et al*. 2000; Koulakov *et al*. 2002; Wang *et al*. 2013) and neuronal integration (Aksay *et al*. 2007). The cortical networks are continuously active and remember different stimuli by transient changes in firing rates. In contrast, the recordings from tadpole reticulospinal hdIN neurons suggest that, when the tadpole is at rest, the hindbrain neurons responsible for the summating excitation (hypothesised in our modelling as excitatory ‘extension’ neurons: hexNs) are silent and do not excite them. Instead, the ‘extension’ neurons remember any transient sensory input by firing for a short period of time. These neurons also ensure that the same stimulus can produce different response delays because their firing pattern, like the timing of EPSPs in the hdINs, is different from trial to trial. This is what is seen in real animal movement responses.

Preliminary extracellular and whole‐cell recordings have revealed hindbrain neurons with suitable firing in response to skin stimuli (James, 2009; Koutsikou *et al*. 2016). The next goal is to define the anatomical identity of these hindbrain extension neurons and use paired recordings to obtain evidence on their synaptic connections with each other and with reticulospinal hdINs. If their probability of making synapses is low, as suggested by our model, this task will be very challenging.

Our present interpretation of the evidence focuses on a recurrent network to explain the persistence of EPSPs to hdINs. Other cellular explanations such as plateau firing (Viana Di Prisco *et al*. 1997) remain possible but while there is no evidence in the tadpole for plateaus (e.g. Li *et al*. 2006), there is a clear precedent for recurrent excitation: in the positive feedback synapses between reticulospinal hdINs that are key to maintaining swimming (Li *et al*. 2006). Lesions to disconnect the midbrain establish that some of the prolonged excitation of hdINs leading to swimming comes from neurons in the hindbrain where preliminary recordings show neurons with suitable firing patterns. Recurrent networks on each side of the hindbrain are therefore a reasonable proposal. However, without evidence on the location, number, properties and synaptic input and output connections of hexNs, further, more detailed, exploration of the recurrent network seems premature and would be highly speculative.

### Why is the decision making process slow?

In motor networks with antagonistic halves coupled by reciprocal inhibition, like the tadpole swimming pattern generator, modelling studies have shown that the two sides can fire reliably in synchrony as well as in alternation (Wang & Rinzel, 1992). During synchrony, each side escapes inhibition by firing quickly before inhibition arrives from the other side. Synchronous firing of the two sides can occur in the tadpole (Li *et al*. 2014) and model tadpole networks show that it is more common when both sides receive similar, fast‐rising excitation (Wang & Rinzel, 1992; Li *et al*. 2014; Roberts *et al*. 2014). It is hard to see how this type of motor output serves a useful function. Synchrony may be avoided by slowing down the excitation reaching the hdINs following sensory stimulation and making it unequal on each side (Buhl *et al*. 2015). Adding noisy synaptic excitation to each side could also reduce the probability of excitation bringing the hdIN populations to spike threshold at the same time and in this way avoid synchronous firing.

What other advantages are there to making the sensory input slow and noisy? Slowing and extending the depolarisation of reticulospinal neurons will provide a period of about a second for the excitation from one skin sensory pathway to sum and be integrated with other sensory inputs. During this period, two or more subthreshold skin stimuli given to any points on the body surface could sum to bring the hdINs to threshold and initiate swimming through spatial summation. Repeated subthreshold stimuli to the same skin sensory location could also reach threshold by temporal summation. Summation of inputs could also occur across different modalities of sensory input like somatosensory and the pineal eye, which is excited by dimming (Jamieson & Roberts, 1999). The duration, noise and delay in the excitation reaching hdINs gives the animal time to ‘consider’ or integrate its response with other inputs or states. Adding synaptic noise will also contribute to variability in response times. This will make responses less predictable, which may be an important contributor to predator evasion particularly in a small and vulnerable animal (Domenici *et al*. 2011).

### Conclusion

In young *Xenopus* tadpoles, reticulospinal hdINs act as accumulator neurons where excitation resulting from sensory input sums noisily to threshold to initiate swimming. This is functionally similar to the integrator role that Mauthner neurons in fish and giant neurons in crayfish play for rapid escape swimming (Edwards *et al*. 1999; Korn & Faber, 2005). However, there are extra steps. Short latency, brief and reliable firing in the sensory pathway response to a transient stimulus arrives in the brainstem where it is extended by currently unidentified neurons. The firing of these neurons introduces noise and unpredictability. They provide a simple form of sensory memory of the stimulus which lasts for around a second. It is remarkable that inserting noise into the sensory pathway was actually predicted on theoretical grounds to account for features of the initiation of eye movements in humans (Carpenter & Williams, 1995). We argue that stretching out the input signal and making the decision process slow and noisy, may allow integration with other sensory inputs and help to produce coordinated movement by avoiding the antagonistic left and right sides of the body contracting together. In older and more advanced animals, the decision to move is correlated with neuron firing in cortical motor centres (Smith *et al*. 2011), which probably influence pathways leading to hindbrain reticulospinal neuron integrators. The finding of equivalent processes in the brainstem of such a young nervous system as the tadpole's points to their fundamental importance when any animal decides to make a coordinated movement.

## Additional information

### Competing interests

The authors declare no competing financial interests.

### Author contributions

The experimental work was carried out in the laboratories of S.R.S., A.R. and R.B. Conception and/or design of the work – S.R.S., A.R. Acquisition, analysis or interpretation of data – S.K., R.M.H., E.B., A.F., W.C.L., R.B., S.R.S., A.R. All authors contributed to drafting and/or revising the manuscript. All authors have approved the final version of the manuscript and agreed to be accountable for all aspects of the work.

### Funding

The Biotechnology and the Biological Sciences Research Council grants: BB/L002353/1, BB/L00111X/1 and BB/L000814/1.
